# Claims-Based Network Analysis of Disease Progressions in Complex and Non-Complex Older Adults

**DOI:** 10.1093/geroni/igab046.2523

**Published:** 2021-12-17

**Authors:** Peter Nicholas Onglao, Ciara Janer, Maria Eloisa Ventura, Lauren Bangerter

**Affiliations:** 1 OptumLabs, Minnetonka, Minnesota, United States; 2 OptumLabs, Eden Prairie, Minnesota, United States

## Abstract

Older adults are the fastest growing subset of complex patients with high medical, behavioral, and social needs. Understanding differences in disease progression patterns between complex and non-complex older adults is critical for understanding disease risk and tailoring patient-centered interventions. We identified complex patients as those having frequent medical encounters and multiple chronic conditions within the first year of the study period and non-complex patients as the converse. This study compares the disease progression patterns of (a) complex and (b) non-complex older adults by creating disease progression networks (DPN) from claims data of 762,362 patients (mean age = 73) from 2016 to 2020. We characterized the network size and density between the complex patient DPN (C-DPN) and non-complex patient DPN (NC-DPN), and compared disease progression incidence, time-to-progression, and age- and gender-related risk. Results show that the C-DPN was denser and had a wider range of values for risk of progression compared to the NC-DPN. This implies more varied disease progression patterns occurring in the complex adults. We were also able to compare (median) time-to-progressions of diseases relative to each subpopulation and found variation in disease progression time. Furthermore, k-means clustering on the network allowed us to identify highly connected diseases involved in many disease pathways that are prevalent among older adults. (e.g., lipoprotein disorders, hypertension, major depressive disorder). Our results suggest that DPNs can be used to identify important conditions and time-points for tailoring care to the complex and non-complex older adults.

